# How Does *Bacillus thuringiensis* Crystallize Such a Large Diversity of Toxins?

**DOI:** 10.3390/toxins13070443

**Published:** 2021-06-26

**Authors:** Guillaume Tetreau, Elena A. Andreeva, Anne-Sophie Banneville, Elke De Zitter, Jacques-Philippe Colletier

**Affiliations:** Univ. Grenoble Alpes, CNRS, CEA, Institut de Biologie Structurale, F-38000 Grenoble, France; guillaume.tetreau@gmail.com (G.T.); Elena.Andreeva@ibs.fr (E.A.A.); Anne-Sophie.Banneville@ibs.fr (A.-S.B.); Elke.De-Zitter@ibs.fr (E.D.Z.)

**Keywords:** pore-forming toxin (PFT), pesticidal protein, bacteria, crystals, crystalline formulation, bioinsecticide, biotechnology, structural biology

## Abstract

*Bacillus thuringiensis* (*Bt*) is a natural crystal-making bacterium. *Bt* diversified into many subspecies that have evolved to produce crystals of hundreds of pesticidal proteins with radically different structures. Their crystalline form ensures stability and controlled release of these major virulence factors. They are responsible for the toxicity and host specificity of *Bt*, explaining its worldwide use as a biological insecticide. Most research has been devoted to understanding the mechanisms of toxicity of these toxins while the features driving their crystallization have long remained elusive, essentially due to technical limitations. The evolution of methods in structural biology, pushing back the limits in size of amenable protein crystals now allows access to be gained to structural information hidden within natural crystals of such toxins. In this review, we present the main parameters that have been identified as key drivers of toxin crystallization in *Bt*, notably in the light of recent discoveries driven by structural biology studies. Then, we develop how the future evolution of structural biology will hopefully unveil new mechanisms of *Bt* toxin crystallization, opening the door to their hijacking with the aim of developing a versatile in vivo crystallization platform of high academic and industrial interest.

## 1. Introduction

**Figure 1:**
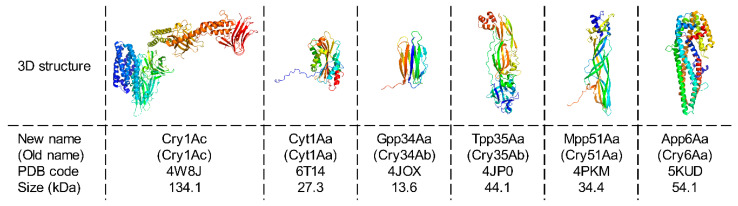
*Bt* can crystallize toxins belonging to six structurally different groups. The structure of a representative toxin is shown for each group: Cry1Ac [10], Cyt1Aa [11], Gpp34Aa (Cry34Ab) [12], Tpp35Ab (Cry35Ab) [12], Mpp51Aa (Cry51Aa) [13], and App6Aa (Cry6Aa) [14]. Each group exhibits structural peculiarities. In the “Cry” family, the core toxin is composed of three domains (two composed uniquely of α-helices and one of β-strands) supplemented (e.g., Cry1Ac) or not (e.g., Cry11Aa) by a C-terminal crystallization domain (see Section 3 for more details). In the “Cyt” family, toxins are formed by a central β-sheet surrounded by two α-helices hairpins. “Gpp” toxins are all-β structured proteins with low molecular weight. “Tpp” and “Mpp” toxins are elongated proteins essentially composed of β-strands containing domains characteristics of the Toxin_10 (Bin-like, Pfam #PF05431) and ETX/MTX2 families (Pfam #PF03318), respectively. In contrast, the “App” family encompasses elongated toxins predominantly composed of α-helices. Thus far, only structures of a few toxins from the “Cry”, “Cyt”, and “Tpp” families have been directly solved using in vivo-grown nanocrystals while the others were obtained thanks to in vitro recrystallized activated and/or full-length toxins (see Section 6 for more details). 3D structures are represented in ‘cartoon’ mode and colored from blue (N-terminal) to red (C-terminal) using PyMOL Molecular Graphics System version 2.1.1. It has recently been suggested the use of the term “toxins” be avoided and that these proteins be be described as “pesticidal proteins” [3]. Although we support this initiative, we use the term “toxins” throughout this manuscript to comply with the journal name and for practical reasons. New toxin names are used in the manuscript to support the new proposed nomenclature and when different, old names are systematically given under brackets to facilitate the link with previous studies. ‘Old names’ and ‘new names’ refer to the nomenclature established by Crickmore et al. in 1998 [15] and in 2020 [3], respectively.

**Figure 2:**
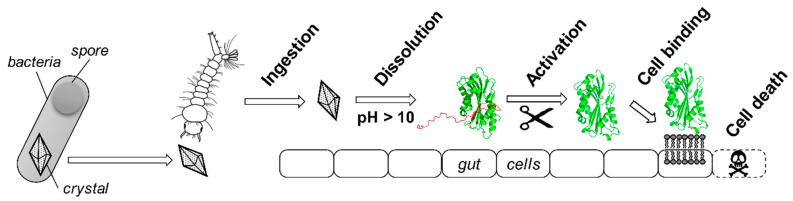
Schematic mode of action of *Bt* toxins. Most *Bt* toxins share a similar multistep mode of action [16]. They are produced as crystals during the sporulation of the bacterium. Spores and crystals are released in the aqueous environment where they are ingested by an insect larva (here, a mosquito larvae) The crystal then specifically dissolves due to the highly alkaline pH of the insect gut to release a protoxin. The propeptide located in the N- and/or C-terminal part of the protein (here, in red) is then cleaved off by digestive enzymes to release a protease-resistant mature toxin core (here, in green). This activated toxin can bind to protein receptors present at the surface of the gut cell membranes or directly interact with lipids, depending on the toxin, to oligomerize and perforate the membrane, ultimately leading to gut cell disruption and insect death. The protoxin and toxin represented here are both from Cyt1Aa produced by *Bt* subsp. *israelensis* (*Bti*), with PDB accession numbers 6T14 [11] and 3RON [17], respectively. 3D structures are represented in ‘cartoon’ mode using PyMOL Molecular Graphics System version 2.1.1.

*Bacillus thuringiensis* (*Bt*) is a natural crystal maker that has evolved over millions of years to form a set of hundreds of subspecies, each crystallizing one or more toxins required for infecting insect larvae [1]. *Bt* is able to produce a high diversity of toxins belonging to nine structurally different categories, of which six are produced as crystalline inclusions during *Bt* sporulation (Figure 1) [2,3,4]. Each *Bt* subspecies is specific to an invertebrate group, which is determined by the particular cocktail of crystalline toxin(s) that it expresses [5]. This explains why it is the most used biological insecticide worldwide to control different insect species considered as agricultural pests (e.g., caterpillars or mealworms) or as threats for human health (e.g., mosquitoes) [6]. Despite strong structural differences (Figure 1), all *Bt* toxins have a similar mode of action and their crystals exhibit shared properties (Figure 2). Indeed, the purpose of toxin crystallization is for *Bt* to ensure the long-term storage of its main virulence factors in an aqueous environment so that they remain functional until they are ingested together with the spores by the insect host. The release of toxins is controlled by the specific dissolution of the crystals at high pHs only (>10), which correspond to the insect intestinal pH and further participate to *Bt* host specificity. Altogether, this shows that *Bt* has developed a wide array of strategies to achieve similar crystal formation and dissolution properties for proteins of drastically different sequences and structures. Considering that the biology and ecology of *Bt* has been extensively studied for decades and that several molecular tools have been developed to enable the recombinant production of proteins in *Bt* [7,8,9], one could wonder whether the crystallization machinery of *Bt* can be hijacked to produce crystalline formulations of proteins of interest for structural biology and biotechnological applications. 

This two-articles series aims at addressing the following question: can *Bt* be turned into a custom crystal biofactory and how? A prerequisite for using *Bt* to produce custom microcrystals is to characterize the mechanisms allowing *Bt* to crystallize such a wide variety of toxins. This topic has already been addressed several times in excellent comprehensive review articles, which we invite the reader to explore for more detailed information on specific aspects [18,19,20,21,22,23]. In this review, we centralize the most relevant information in regard with the general question of this series by adopting a comparative approach between in vitro and in vivo crystallization processes to highlight how *Bt* can control the specific crystallization of a large set of diverse toxins while sustaining the production of many membrane and soluble proteins involved in spore formation [24,25]. Most notably, we develop the diversity of the mechanisms used to produce a high quantity of toxins (Section 2), the intrinsic crystalline characteristics of toxins (Section 3), the role of accessory proteins (Section 4) and eventually how a fine equilibrium between these three factors conditions toxins crystallization in *Bt* (Section 5). Then, we conclude by exploring the past, current and future contribution of structural biology accompanying its (r)evolution (Section 6). The aim of this article is thus to provide the necessary knowledge on the *Bt* system for a large range of multidisciplinary readers to understand the relevance and feasibility of the main question addressed.

## 2. A Diverse Set of Mechanisms to Produce High Quantities of Toxins in *Bt*

The tremendous quantity of toxins produced by *Bt*, whose crystalline inclusion can represent up to 40% of the sporulated cells dry weight [9,19], is essentially achieved by a fine and timely regulation of toxin gene transcription and by a stabilization of toxin transcripts [19,20]. 

*Bt* possesses the same transcriptional regulatory factors—called sigma factors (σ)—that *Bacillus subtilis* (*Bs*) uses to regulate the expression of key genes at different stages of its sporulation [26]. Most genes encoding Cry and Cyt toxins are regulated by σ^E^ and σ^K^ that are active during sporulation [20,22]. The promoter σ^H^, which is active during the transition from the vegetative growth to the sporulation phase, can also be found but it generally contributes little to the overall toxin gene expression [22]. Cry3Aa is a notable exception as it uses σ^A^, active as early as the vegetative phase, for its production [19]. Each toxin gene exhibits a specific combination and arrangement of sigma factors in the 5′ untranslated region (5′ UTR). While sigma factors account for most of the *Bt* toxins gene transcription regulation, other complementary mechanisms exist and have generally only been identified for specific *Bt* strains and/or toxin genes so far [20]. As in all prokaryotes, the initiation of gene transcription in *Bt* relies on ribosome binding sites (RBS) located between the promoter(s) and the ‘start’ codon of the gene [27]. While the ‘GGAGG’ RBS motif is highly conserved in the *Bacillus* genus [28], variations in the length and sequence of the translation initiation region (TIR), which encompasses the RBS region, can induce different secondary structures and strongly modulate the gene transcription level [29]. 

Two major mechanisms stabilizing the mRNA in its 5′ and 3′ ends have been identified in *Bt*, notably explaining why the half-life of *Bt cry* gene products (approx. 10 min) is particularly high [30]. In bacteria, mRNA decay is driven by different RNAses degrading mRNA from its 5′ and/or 3′ end [31]. Many toxin mRNAs are stabilized by repeated inverted sequences at their 3′-end, called stem-loops, forming secondary structures that block the mRNA degradation by 3′-exonucleases [32,33]. Stabilization in 5′ can also be performed thanks to a Shine Dalgarno-like sequence present in 5′ of the coding gene [34]. It is believed that this sequence stabilizes the mRNA by a “ribosome stalling” mechanism [35], promoting the recruitment of the 30S ribosomal subunit thanks to its sequence homology with the 3′ end of 16S RNA [34]. Once bound, the subunit would protect the mRNA from degradation by 5′ exonucleases, such as RNAse J, extending its half-life and allowing more toxin to be produced from a single mRNA [19,32,35]. Such a motif has initially been identified upstream of *cry3aa* gene but it seems more widespread as it is also present in other *cry3* genes in *Bt* and in genes from other Gram-positive bacteria [19]. 

## 3. Toxins Evolved to Be Crystallization-Prone

Expressing a very high quantity of proteins is not sufficient to make an ordered assembly of protein leading to a crystal; instead, it generally leads to inclusion bodies with little intrinsic organization [36]. The *Bt* crystal properties—i.e., stability in aqueous environment, solubility restricted to high pHs only and protease-resistant core protein—are driven by the toxin itself and its crystallization properties, which imposes the toxin’s structural characteristics. 

**Figure 3:**
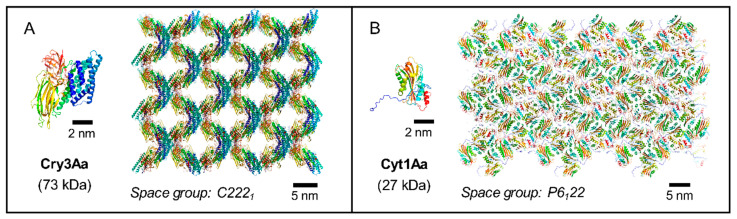
Different *Bt* toxins with different structures exhibit different crystallization strategies to achieve similar crystal features. Here two examples are shown of toxins structures solved directly from crystals grown in *Bt*. (**A**) The stacking of the three-domain Cry3Aa protoxins allows generating solvent channels of 3–5 nm width that traverse the crystal cell along the *c* axis. The same crystal packing is observed in in vivo [37] and in in vitro crystals [38]. (**B**) In vivo crystallization of Cyt1Aa is essentially driven by its homodimerization through the domain-swapping of the N-terminal propeptides [11], which differs from the crystallization of the activated Cyt1Aa toxin in vitro [17]. 3D structures of Cyt1Aa (PDB accession number: 6T14 [11]) and Cry3Aa (PDB: 4QX0 [37]) protoxins are represented in ‘cartoon’ mode and colored from blue (N- terminal) to red (C-terminal) using PyMOL Molecular Graphics System version 2.1.1.

The toxin Cry3Aa, for example, crystallizes through a checkerboard-like packing into crystals with high solvent content (Figure 3A) [37]. Its dissolution is restricted to high pH by four intermolecular salt bridges located at contacts with three different neighboring molecules [38]. Interestingly, Cry3Aa protoxin is able to crystallize both in vivo and in vitro and it exhibits the same crystal-packing interfaces, highlighting that the crystallization process of this toxin is essentially, if not exclusively, driven by intrinsic protoxin characteristics [37,38,39]. 

However, this is not a feature common to all *Bt* toxins as some are unable to crystallize outside the cell in their protoxin form, such as the 27 kDa Cyt1Aa (Figure 3B). Indeed, only the protease-resistant core protein of Cyt1Aa could be recrystallized in vitro after the N-terminal propeptide had been proteolytically removed from the toxin [17]. The Cyt1Aa protoxin structure directly solved from in vivo crystals allowed to elucidate the reason behind this phenomenon [11]. During the sporulation of the *Bt* cell, crystallization of Cyt1Aa is initiated by a domain-swapping of the N-terminal propeptide of two nascent Cyt1Aa monomers, leading to the formation of a domain-swapped dimer. This interface drives the crystallization of Cyt1Aa and it has the largest buried surface area (BSA) in the crystals. The dissolution of Cyt1Aa crystals relies on the dissociation of this dimer by electrostatic repulsion between residues around the domain swapping area [11]. Recrystallizing the protoxin after crystal dissolution would require the swapping of the N-terminal propeptide to be reconstituted, which corresponds to a high free-energy barrier hardly reachable in vitro [40]. This could explain why only activated Cyt1Aa toxins devoid of N-terminal propeptides could be recrystallized in vitro and why the crystal packing differs between in vivo crystals of protoxin and in vitro crystals of toxin [11,17]. Intriguingly, the homologous non-cytolytic Cyt2Ba protoxin, which exhibits a similar 3D structure and shares 33% of sequence identity with Cyt1Aa, could crystallize in vitro with a crystal packing driven by a N-terminal domain-swapping similar to Cyt1Aa in vivo crystals [41]. This might be linked to the involvement of a chaperone for Cyt1Aa crystallization, which is absent for Cyt2Ba (see Section 4 for further discussion).

*Bt* toxins of 130-140 kDa, comprising the anti-lepidopteran Cry1 groups of toxins, rely on yet another crystallization mechanism [18]. These toxins possess a C-terminal domain—generally referred to as ‘crystallization domain’—which represents approximately half of the protoxin size, is highly conserved among 130–140 kDa toxins and is mandatory for their crystallization [18]. While the N-terminal core toxin is devoid of cysteines, the C-terminal domain contains 14–19 cysteines located on flexible loops that form intra- and intermolecular bonds contributing to the formation and/or stabilization of the crystal [10,18,42]. However, not all cysteines are of equal importance. Full-length Cry1Ac contains a total of 16 cysteines, 14 of which are located in the C-terminal domain while the two others are on the short N-terminal propeptide. When produced in *E. coli*, purified Cry1Ac protoxins tend to rapidly form hardly soluble aggregates through intermolecular disulfide cross-linking [10,19]. Mutation of the 14 cysteines from the C- terminal domain led to a more stable protein in solution, which could form crystals in vitro with similar crystal packing as in vivo-grown crystals [10,43], suggesting that these cysteines have some role in protein–protein interactions but do not drive the crystallization. However, when all 16 cysteines were mutated, crystals formed in vivo promptly solubilized after sporulation, indicating that the two cysteines from the N- terminal propeptide play a major role in the protoxin crystal stability outside the cell, although the exact mechanism is yet to be determined [10,18]. Structures directly determined from in vivo-grown crystals are still lacking for investigating further the role of these cysteines in Cry1 crystal formation and dissolution pathways. 

## 4. The (Facultative?) Role of Accessory Proteins

Crystallization of *Bt* toxins sometimes requires the involvement of additional proteins. They have been coined with many different names including ‘chaperone-like proteins’ [44], ‘molecular chaperones’ [45], ‘crystallization proteins’ [19], ‘helper proteins’ [46,47], or even ‘accessory proteins’ [48]. Obviously, this plethora of appellations has been more misleading than informative. Indeed, the fact that their absence (sometimes) leads to the absence of a crystal does not necessarily mean that they are directly and actively involved in the crystallization process. They may act at different levels—e.g., they can stabilize the protein and/or protein–protein interactions, help protein folding or increase its concentration—which both help to achieve the required conditions for crystallization without actually directly facilitating or participating in crystal contacts. Therefore, one should be careful with the terminology employed. In this part and elsewhere in the article, we use the term ‘accessory proteins’ which encompasses all the proteins directly or indirectly implicated in *Bt* toxin crystallization as it provides no a priori information on the mechanism involved, which is sometimes unknown or unclear. 

These accessory proteins are generally organized in an operon together with *Bt* toxin gene(s) [18,19]. The operons of several 55–65 kDa toxins have, downstream of the toxin-coding gene, a second open reading frame (ORF2) with high sequence homology to the ‘crystallization domain’ of the 130–140 kDa toxins (discussed in Section 3). A series of experiments fusing the ORF2 of Cry19A with the Cry19A toxin or with the N-terminal core toxin domain of Cry1C both led to the formation of inclusions [49]. This suggests that ORF2 might play the same role as the ‘crystallization domain’ of 130–140 kDa toxins through a similar, albeit still poorly understood, mechanism [18,20]. The efficiency and universality of such a domain/ORF2 might explain why it is so widespread in many different *Bt* subspecies for a number of different toxins within the Cry structural group [3,18].

Another kind of accessory protein called P29 (due to its 29 kDa size) is found in the operon of Cry2Aa toxin and contains a 15-residues motif repeated eleven times [50]. It has been hypothesized that it would form a framework favoring the nucleation of Cry2Aa toxins as they are synthesized, driving their assembly into characteristic cuboidal crystals [51,52]. While repeat sequences have been identified in the C-terminal region of other toxins, their exact role in crystallization is still unknown [18].

P20, a protein of 20 kDa, is encoded by the third ORF in the Cry11Aa operon from *Bt* subsp. *israelensis* (*Bti*) and it has been shown to be mandatory for the crystallization of Cyt1Aa [53], whose coding gene is located in the neighboring operon upstream Cry11Aa’s one in the pBToxis megaplasmid [54]. The structural modelling of P20 revealed that it exhibits a high structural homology with the activated form of Cyt1Aa [55]. Knowing that P20 forms a transient complex with the nascent Cyt1Aa peptide chain [56], one could hypothesize that it offers a scaffold for the setting of the domain-swapping of the Cyt1Aa N-terminal domain [11], in line with the “heritable template” hypothesis [57]. Interestingly, *Bt* subsp. *medellin* possesses a homolog of P20—namely P21—located in 5′ of a homolog of Cyt1Aa—namely Cyt1Ab—with whom they share 75% and 86% identity, respectively [58]. This would further argue in favor of a co-evolution between these accessory proteins and their Cyt1 counterpart. Interestingly, while the non-cytolytic Cyt2Ba can crystallize in vivo and in vitro through a similar domain-swapping strategy, it does not involve any P20-like protein [41]. Intriguingly, P20 can stabilize and/or help the crystallization of a wider range of toxins from the same *Bt* subspecies (e.g., Cry4Aa [59], Cry11Aa [60]) but also from other subspecies (e.g., Cry1Ac [46], Cry2Aa [51], Cry3Aa [44]), suggesting that it exhibits. at least in part, universal role(s).

## 5. Crystallization in Bt Is a Finely-Tuned Multifactorial Process

Each of the three factors presented above (i.e., the finely-regulated production of high quantities of toxins, their crystallization proneness, and the facultative presence of accessory proteins) rarely solely drive toxin crystallization [18,19,20]. They are highly toxin-dependent, meaning that they might be neither universal nor mandatory, and while they can exhibit complementary functions, they are not mutually exclusive. 

For example, Du et al. demonstrated that crystals of Cry1 toxins obtained in vivo by *Bt* and in vitro after denaturing treatment with urea both formed bipyramidal crystals, indicating that the shape and size of crystals were driven by the toxin itself [61]. However, the pH at which they dissolved strongly differed, i.e., 9.5–10.5 vs. 12 for in vivo and in vitro crystals, respectively, which supports that some key protein–protein interactions—notably involving disulfide bonds—that drive crystal dissolution can be stably established only within the bacterial cell [19]. This contrasts with Cry3Aa that can crystallize in vivo and in vitro while conserving similar features [37,38] and with Cyt1Aa for which the protoxin is unable to recrystallize in vitro [17], notably due to contrasting crystal packing strategies between the protoxin and the toxin [11].

As discussed above (see Section 2), Cry3Aa production is controlled by a sigma factor (σ^A^) active at the vegetative stage and its mRNA is stabilized by the STAB-SD sequence [34]. It was however shown that the sporulation promoters of Cyt1Aa (σ^E^ and σ^K^ [22]) were sufficient to compensate for the absence of both σ^A^ and STAB-SD to produce similarly shaped and sized crystals of Cry3Aa [62]. However, when Cyt1Aa promoters were combined with STAB-SD, this allowed a significant increase in yield of Cry3Aa produced and the size of the crystal, but this came with high fitness costs resulting in a reduced number of spores produced [62]. STAB-SD has been used to increase the yield and subsequent crystal size of different toxins with a large variation in the outcome, with an order of magnitude between the lowest (e.g., for Cry2Aa [49,63] and Cry11Aa [63]) and the highest increase (e.g., Cry11Ba [64], Cry19Aa [49], Mpp60Aa and Mpp60Ba—formerly known as Cry60Aa and Cry60Ba, respectively [65]). This exemplifies how different factors affecting similar parameters (here, the number of proteins produced per transcript by increasing its number and/or by stabilizing it) can complement themselves and that additional toxin-dependent factors seem to regulate the relative contribution of each factor, notably to balance the energetic needs for the concomitant formation of the crystal and the spore. Determining the role of each factor is therefore necessary but insufficient to understand the whole crystallization process. Notably, more crystallographic studies directly using crystals grown in *Bt* are required to widen our understanding of the underlying crystallization mechanisms, in addition to providing key information on the toxin’s mode of action.

## 6. The (R)Evolution of Structural Biology Allows the Unraveling of Key Steps in the Crystallization Pathways of *Bt* Toxins

**Figure 4:**
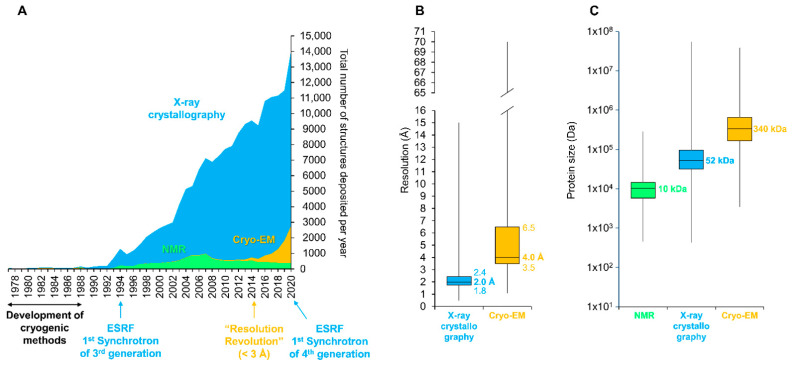
Participation of the three major structural methods in the determination of protein structures. (**A**) Yearly evolution of protein structure deposition on the RCSB Protein Data Bank (PDB, [70]). Key milestones are indicated below the graph. (**B**) Box plot representation of the resolution (in Angstroms (Å)) is indicated for X-ray crystallography and Cryo-EM. (**C**) Box plot representation of the protein size (in Dalton) is indicated for NMR, X-ray crystallography, and Cryo-EM. For the entire figure, data related to X-ray crystallography, Nuclear Magnetic Resonance (NMR), and cryo-electron microscopy (Cryo-EM) are indicated by blue, green, and orange colors, respectively. ESRF, European Synchrotron Radiation Facility (Grenoble, France). Data were retrieved from the RCSB database on 31 January 2021 (https://www.rcsb.org).

Determining the structure of a protein is a key milestone in deepening the study and understanding of its function. Among all the methods available in structural biology, macromolecular crystallography (MX) remains the favored one for determining the structure of proteins at high resolution (Figure 4) [66]. It is notably due to the eased access to finely tuned and reliable MX beamlines at synchrotron sources that this technique has been used to solve >90% of the structures present in the Protein Data Bank (PDB). In synchrotrons, diffraction data are generally collected from a single macro-crystal (10–100 µm) maintained in cryogenic conditions to collect a complete dataset by oscillation methods, yielding structures with mitigated radiation damage [67,68]. Unfortunately, the submicrometer size of the crystals naturally produced by *Bt* has long hindered their study, as they were not amenable to structure determination by conventional oscillation methods at synchrotron sources. This is because the progression of X-ray-induced radiation damage is only delayed but not eliminated by flash-cooling. In practice, this means that the smaller the crystals, the more radiation sensitive they are, requiring more crystals to obtain a radiation damage-free dataset [69]. 

Investigation of the link between structural features and *Bt* toxin function had to rely on direct and/or indirect approaches to deal with the *Bt* crystal size limitation. A direct approach consisted in the solubilization of the natural *Bt* toxin crystals in alkaline buffers before toxins were recrystallized in vitro onto bigger crystals compatible with MX beamlines of synchrotrons. Although a restricted number of toxins could recrystallize in their full-length, such as Cry3Aa [38], Gpp34Aa (Cry34Ab) [12], Tpp35Ab (Cry35Ab) [12], and Cyt2Ba [41], most *Bt* toxins are reluctant to recrystallization in vitro [4]. This can be attributed to the need of accessory proteins, of the specific intracellular environment and/or of particular toxin–toxin interactions driven by propeptides during the early stages of protein production [18,19]. To circumvent this problem, the most adopted strategy consisted in mimicking the toxin activation step within the insect gut by proteolytically activating the full-length toxin in vitro to release the core toxin devoid of its N- and/or C-terminal parts. By doing so, several structures of activated *Bt* toxins were obtained, providing key information on the domains and amino acids involved in insect receptor recognition, oligomerization, and cell perforation, notably driving point-mutation strategies to improve toxin efficacy and host spectrum [71]. However, they offered a limited view of the mechanism of crystallization within the cell, as the crystal packing of activated toxins may differ from the packing of full-length toxins in *Bt* cells [11]. 

The absence of structures for most toxins prompted scientists from the field to embrace indirect strategies to investigate the implication of given amino acid(s) in the function of the toxins. Some used a mutation-based approach, which consists of the mutation of a selected set of amino acids considered as important, based on the sequence, on domain prediction and on homology of the protein sequence with other known *Bt* toxins. Then, the effect of these mutations on the toxin activity and capacity to form crystals can be observed [72,73,74]. Tridimensional homology modelling the term ‘homology modelling’ summarizes it all; I therefore suggest removing the highlighted part of the sentence was also conducted to explore the interactions between toxin domains and potential insect receptors [55,75,76]. Although informative, they both offer a restricted view on the implication of a limited set of amino acids lacking most of the interactions that can drive the toxin function as those cannot be easily extrapolated from the protein amino acid sequence. 

Recent advances in crystallography methods allowed the unraveling of some hidden features driving the in vivo crystallization process of a few *Bt* toxins. A major breakthrough has been the introduction of serial crystallography—whereby diffraction data are collected from a myriad of still crystals instead of a single crystal oscillating in the X-ray beam—at X-ray free electron lasers, whose peak brillance is fifteen orders of magnitude higher than synchrotrons, resulting in the delivery of the same amount of photons in a ~40 fs pulse, as the latter would produce in 1–10 ms [77]. This method is generally limited to a restricted number of proteins able to crystallize easily in vitro due to the high amount of protein needed for performing an experiment (from 0.05 to 1 gram, depending on the experimental goal). In the case of *Bt*, its proneness to produce normalized, micro-meter sized crystals in laboratory settings, the stability of its crystals in water, and the availability of many procedures for crystal purification made it perfectly adapted for such an approach. This led to the determination of the structures of the full-length toxins Cry3Aa [37] and Cyt1Aa [11] from *Bt*, but also of the binary toxin Tpp1Aa/2Aa (formerly BinAB) from *Lysinibacillus sphaericus* produced in *Bt* [78]. This notably allowed confirming the moieties that support the crystallization-proneness of Cry3Aa [37,38] and the unveiling of the key role played by the N-terminal propeptide of Cyt1Aa during its in vivo crystallization [11,17]. The opening of new MX beamlines at operating XFELs and the will of such facilities to tend toward a more user-friendly platform hold promise that access to these facilities will become less competitive in the future, enabling more experiments to be performed [77]. Moreover, the success of serial crystallography at XFELs inspired investigators to import serial crystallography at synchrotrons (SSX) [79,80,81,82,83]. With the upgrade of synchrotrons to “extremely brilliant sources” (EBS, Figure 4), resulting in a 10^1^–10^3^ fold increase in flux at microfocus beamlines [84], and the installation at all synchrotron facilities of dedicated serial crystallography beamlines, SSX experiments are on the edge of democratization, expanding the possibilities for *Bt* studies. 

In parallel to the development of crystallography, cryo-electron microscopy (cryo-EM) also experienced a revolution, pushing back the limits of resolution attainable (Figure 4) [85]. Using this technique, the structures of the secreted soluble Vip3Aa and Vip3Bc toxins were determined, providing key information into their activation, oligomerization, and membrane perforation processes [86,87]. Hence, the forthcoming years could allow a significant number of structures of native *Bt* toxins to be solved, significantly expanding our understanding of the diversity of toxin crystallization strategies in *Bt*.

## 7. Conclusions

*Bacillus thuringiensis* has developed a number of strategies to crystallize a large range of toxins with contrasting tridimensional structures, including a highly regulated toxin expression, the production of accessory proteins, and intrinsic toxin crystallization properties. Decades of research have allowed the unraveling of some of the key factors driving the crystallization of several *Bt* toxins. However, the lack of structures of full-length toxins directly determined from crystals produced in vivo has long limited the depth of investigation of *Bt* toxin crystallization. Recent advances in structural bology initiated the understanding of as yet elusive crystallization mechanisms. Current and future development in cryoEM and in serial crystallography at X-ray free electron lasers and synchrotrons should enable to expland our knowledge on Bt toxins and on the mechanisms they exploit for in vivo crystallization.

## Data Availability

Not applicable.
