# Erratum: Tetreau et al. How Does *Bacillus thuringiensis* Crystallize Such a Large Diversity of Toxins? *Toxins* 2021, *13*, 443

**DOI:** 10.3390/toxins13090598

**Published:** 2021-08-27

**Authors:** Guillaume Tetreau, Elena A. Andreeva, Anne-Sophie Banneville, Elke De Zitter, Jacques-Philippe Colletier

**Affiliations:** Univ. Grenoble Alpes, CNRS, CEA, Institut de Biologie Structurale, F-38000 Grenoble, France; guillaume.tetreau@gmail.com (G.T.); Elena.Andreeva@ibs.fr (E.A.A.); Anne-Sophie.Banneville@ibs.fr (A.-S.B.); Elke.De-Zitter@ibs.fr (E.D.Z.)

The authors wish to make the following corrections to this paper [1]. 

The eleventh sentence of Figure 1 caption should read as follows: “It has recently been suggested that the use of the term “toxins” be avoided and that these proteins should be described as “pesticidal proteins” [3].”

The seventh sentence of paragraph 4 in Section 4 should read as follows: “Intriguingly, P20 can stabilize and/or help the crystallization of a wider range of toxins from the same *Bt* subspecies (e.g., Cry4Aa [59], Cry11Aa [60]) but also from other subspecies (e.g., Cry1Ac [46], Cry2Aa [51], Cry3Aa [44]), suggesting that it could play a more general role.”

The fourth sentence of Figure 4 caption should read as follows: “(**B**) Box plot representation of the resolution limit (in Angstrom (Å)) for X-ray crystallography and Cryo-EM.”

The fourth sentence of paragraph 3 in Section 6 should read as follows: “Tridimensional homology modelling was also conducted to explore the interactions between toxin domains and potential insect receptors [55,75,76].”

The fifth sentence in Section 7 should read as follows: “Current and future development in cryoEM and in serial crystallography at X-ray free electron lasers and synchrotrons should enable to expand our knowledge on Bt toxins and on the mechanisms they exploit for in vivo crystallization.”

The errors were introduced during authors writing. Authors apologize for any in-convenience caused to the readers by this change. The change does not affect the scientific results. The original version of this manuscript will be updated soon.
